# Lipid and lipoprotein metabolism in microglia: Alzheimer’s disease mechanisms and interventions

**DOI:** 10.1016/j.jlr.2025.100872

**Published:** 2025-08-11

**Authors:** Kayla G. Sprenger, Emma E. Lietzke, John T. Melchior, Kimberley D. Bruce

**Affiliations:** 1 Department of Chemical and Biological Engineering, University of Colorado Boulder, Boulder, CO, USA; 2 Biological Sciences Division, Pacific Northwest National Laboratory, Richland, WA, USA; 3 Department of Pathology and Laboratory Medicine, University of Cincinnati, Cincinnati, OH, USA; 4 Department of Neurology, Oregon Health and Science University, Portland, OR, USA; 5 Division of Endocrinology, Metabolism, and Diabetes, University of Colorado Anschutz Medical Campus, Aurora, CO, USA

**Keywords:** microglia, lipids, lipoproteins, apolipoproteins, Alzheimer's disease

## Abstract

Alzheimer's disease (AD) presents a significant challenge owing to its widespread prevalence and complex neuropathogenesis, affecting millions worldwide. Current therapeutic strategies that predominantly target amyloid-beta accumulation are insufficient, particularly for ApoE4 carriers. Alterations in lipid composition are well documented in AD, characterized by reductions in phospholipids and sulfatides, along with increases in cholesterol, cholesteryl esters, and triglycerides (TGs). Microglia, the brain's resident immune cells, link dysfunctional lipid processing to AD neuropathogenesis. For example, genetic studies have pointed to microglial lipid and lipoprotein processing gene variants as some of the strongest risk factors for AD. In addition, microglial dysfunction, characterized by lipid droplet accumulation, increased cholesterol and TG levels, and altered lipid transport, may exacerbate the pathological hallmarks of AD, such as amyloid-beta and tau accumulation. Conversely, emerging studies have shown that strategies aimed at inhibiting lipid droplet accumulation in microglia, reducing TG synthesis, and promoting the activity of lipoprotein receptors expressed by microglia can improve cell functions and markers of AD pathology. This review dissects the interplay between microglial lipid metabolism and AD, highlighting the significance of lipid transport and trafficking within the CNS. Given the intrinsic link between microglial metabolism and AD progression, emerging and potential therapeutic strategies aimed at restoring lipid handling and improving microglial function are explored. This review provides a comprehensive examination of the emerging literature, detailing the current state of knowledge on microglial lipid metabolism, its genetic underpinnings, and the potential for novel interventions targeting these mechanisms to ameliorate AD pathology.

Recent estimates indicate that approximately 6.9 million individuals are currently living with Alzheimer's disease (AD) in the United States (1). At a cellular and molecular level, AD is characterized by several pathological features, including neuroinflammation, neurodegeneration, amyloid-beta (Aβ) accumulation, hyperphosphorylated tau protein, neurofibrillary tangles, lipid droplet (LD) accumulation, and cellular dysfunction. Collectively, these processes clinically manifest as cognitive dysfunction, memory decline, and, ultimately, death. Despite the widespread prevalence and devastating nature of AD, effective interventions that prevent AD onset or progression remain largely absent. While there have been significant advancements in our understanding of the neuropathogenesis of AD, current therapeutic strategies primarily target only one aspect of the disease. For instance, recent FDA-approved AD therapies, such as lecanemab, specifically target Aβ accumulation (2). Alhough these treatments have shown some promise in slowing cognitive decline (2, 3), their effectiveness has been inconsistent, and in some individuals (e.g., *APOE4* gene carriers) may even be detrimental (4). This highlights a critical gap in treatment options, underscoring the urgent need for more comprehensive approaches that more broadly address the multifaceted neuropathogenesis of AD.

A promising therapeutic target is brain lipid metabolism, which is intrinsically linked to brain function. This is perhaps unsurprising given that lipids are the main component of brain tissue, making up 78% of the dry weight of myelin and 35–40% of gray matter (5, 6). More than 150 years of cumulative research has demonstrated that the brain is rich in phospholipids (PLs), sphingolipids, and cholesterol. These studies have also shown that the lipid composition of specific cells and brain regions is precisely coordinated, reflecting both structural and functional differences. For example, the human hippocampus is rich in phosphatidylcholine (PC) (7), myelinating oligodendrocytes are rich in sphingolipids, and microglia, the brain resident macrophages, are rich in neutral lipids, such as cholesteryl ester (CE) and triglycerides (TGs) (6, 8). Notably, changes in the lipid composition of the brain have been repeatedly implicated in the neuropathogenesis of AD (for a recent comprehensive review, see Ref. (9)). While complex, these changes involve robust reductions in PLs and sulfatides, yet increased cholesterols and CE, in both human brains and rodent models of AD (9, 10). Interestingly, the cerebellum, which is somewhat protected against AD pathology, lacks the major alterations in lipid composition seen in AD-vulnerable regions (10). Such changes in lipid composition can even be visualized in AD brains. Indeed, when Dr Alois Alzheimer initially described AD, he described three major pathological hallmarks: amyloid-containing plaques, tau-containing neurofibrillary tangles, and areas of lipid accumulation that he referred to as “adipose inclusions” (11). Given the critical nature of brain lipid composition, these findings underscore the importance of understanding the mechanisms linking altered lipid processing to AD pathology and the need to identify new therapeutic targets and interventions that may broadly improve AD outcomes.

In the search for lipid-centric mechanisms that may drive AD, recent studies have highlighted the role of microglia, the key innate immune effector cells of the brain. Over 100 years after Dr Alzheimer reported LD accumulation in AD brains, several groups have shown that LDs predominantly accumulate in microglia (12, 13). Moreover, these LDs are rich in TGs and CEs, known to be elevated in AD brains (12). LD accumulation in microglia may not only reduce the fluidity of these cells, which rely on their motility to perform surveillant and phagocytic functions, but recent studies have also shown that microglial-LDs may actively promote AD pathology by promoting the accumulation of Aβ and phosphorylated tau (12, 14). Findings from these recent studies highlight microglial LDs as a modifiable target to improve AD pathology, but further studies are needed to determine whether the reduction of LDs per se is a viable therapeutic strategy.

Nonetheless, the interaction between microglial lipid metabolism and AD is robustly supported by large-scale genome-wide association studies, which have identified AD risk genes that regulate brain lipid metabolism and are abundantly (but not always predominantly) expressed by microglia (e.g., *APOE*, phospholipase C gamma 2, triggering receptor expressed on myeloid cells [*TREM2*], phospholipase D family member 3, Clusterin/ApoJ [*CLU*], *ABCA7*, Sortilin-related receptor 1, and secreted phosphoprotein 1/osteopontin [*SPP1*]) (comprehensively reviewed in Ref. (15)). Notably, many of these risk genes involve changes to proteins that regulate lipid transport through lipoprotein trafficking. For example, ApoE, CLU, and SPP1 are major protein components of brain-derived lipoproteins (BLps) (16), ABCA7 is involved in the export of cholesterol and PLs to the lipoprotein particle (17), and TREM2 is a promiscuous receptor whose interaction with lipoproteins and apolipoproteins may drive microglial dysfunction in disease (18, 19) (Fig. 1). Together, these findings suggest that microglial lipid and lipoprotein processing may be a rational therapeutic avenue to restore altered brain lipid processing in AD. However, thanks to the recent investments in basic research interrogating the mechanisms underlying AD, there has been a rapid advancement in our fundamental understanding of microglia metabolism and function, brain lipid and lipoprotein transport, as well as the identification of pleiotropic roles of lipid processing factors with otherwise established roles in peripheral metabolism. Therefore, in this review, we evaluate the emerging literature and reexamine inter- and intra-brain lipid transport and intracellular and intercellular lipid trafficking to shed light on the mechanisms by which microglial lipid and lipoprotein metabolism is involved in AD neuropathogenesis, and explore how microglial lipid and lipoprotein metabolism is being targeted to improve the function of microglia and reduce the risk of neurodegenerative disease.

**Fig. 1:**
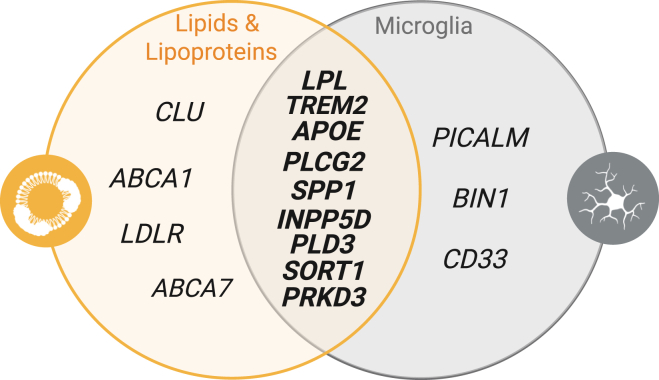
**Venn diagram showing the overlap of AD risk genes involved in lipid and lipoprotein metabolism, which are abundantly expressed by microglia.** ABCA1, ATP-binding cassette sub-family A member 1; ABCA7, ATP-binding cassette sub-family A member 1; AD, Alzheimer’s disease; APOE, apolipoprotein E; BIN1, bridging integrator 1; CD33, cluster of differentiation; CLU, clusterin/ApoJ; LDLR, low-density lipoprotein receptor; LPL, lipoprotein lipase; PICALM, phosphatidylinositol binding clathrin assembly protein; PLCG2, phospholipase C gamma 2; PLD3, phospholipase D family 3; PRKD3, protein kinase D3; SORT1, sortilin 1; SPP1, secreted phosphoprotein/osteopontin 1; TREM2, triggering receptor expressed on myeloid cells 2.

## Microglia: The Nexus of Lipid Metabolism and AD Risk

Microglia are the brain-resident macrophages and key immune effector cells of the CNS. In the typical brain, microglia perform a myriad of essential functions to broadly maintain brain homeostasis, including the phagocytosis of dead cells, debris, and misfolded proteins; the secretion of inflammatory cytokines and other signaling molecules; synaptic pruning; neurogenesis; and the mitigation of infections. Often, microglia are the first cells to respond to a given stimulus, responding rapidly to a variety of environmental cues. To achieve this, activated microglia undergo phenotypic switching, which necessitates a redirection of substrate utilization to meet the increased energetic demands of performing a diverse array of functions and providing trophic support to other glial cells (e.g., oligodendrocytes, astrocytes) and neurons. The prevailing dogma suggests that during activation, microglia increase glucose utilization (glycolysis), reduce mitochondrial oxidative phosphorylation, and reduce the oxidation of FAs. Such a switch toward a Warburg-like metabolism leads to a quick, yet inefficient, generation of ATP. In the typical brain, it is thought that microglia are metabolically plastic, altering substrate utilization and then returning to a metabolic baseline. However, recent studies suggest that in the AD brain, or preclinical models of AD, microglia undergo “metabolic reprogramming,” where glycolysis remains elevated (20, 21). Notably, the “metabolic reprogramming” of microglia is not as conceptually simple as previously thought and involves a synergistic dysregulation between various metabolic pathways, such as increased fructose production (22, 23), increased de novo lipogenesis (14, 24), and reduced cholesterol efflux (25, 26), consistent with increased LD accumulation in microglia in AD, and rodent models of AD. Overall, in AD, microglia appear to lose metabolic heterogeneity, manifesting as disrupted lipid processing. This notion has been recapitulated by transcriptomic analysis of microglia from rodent models of AD, showing a profound upregulation of microglial subpopulations that abundantly express factors involved in lipid and lipoprotein processing, such as *TREM2*, *APOE*, *SPP1*, and *LPL* (27, 28). Interestingly, the transcriptional signatures of these disease-associated microglia (DAM) exhibit some overlap with LD-associated microglia, which accumulate in the aging murine and human brain (24). In addition, while some studies report robust DAM-like gene signatures concomitant with neutral lipid accumulation (29), not all recent analyses of microglia from human AD brains report upregulated DAM gene signatures or LD accumulation (30).

The inconsistent findings from analyses of human microglia likely relate to differences in genetic background, environmental influences, metabolic health of the individual, and even sex: all factors that alter baseline lipid metabolism and transport. A recent analysis of human microglia derived from multiple neurodegenerative disease conditions, including AD, multiple sclerosis, and Lewy body dementia, reported an enrichment of the microglia subsets that were enriched in transcripts involved in lipid and lipoprotein processing (e.g., phospholipase A2 group VII, macrophage scavenger receptor 1, *LPL*, *APOE*, and *APOC1*) (29). In addition, human-induced microglia transplanted into the brains of chimeric mice (5xFAD-hCSF1) exhibit upregulation of DAM genes (e.g., *APOE*, *SPP1*), genes involved in lipid processing (e.g., *APOC1*), accumulate LDs, and display a signature akin to atherosclerotic foam cells (31). In contrast to in vitro studies, but perhaps consistent with an emerging literature suggesting altered binding to lipid and lipoproteins (19), microglia derived from individuals who carry the AD risk R47H variant of TREM2 (TREM2^R47H^) showed a subtle downregulation of genes associated with lipid-laden foam cell formation and showed reduced LD accumulation (31). Overall, these analyses highlight the nuances in lipid metabolism resulting from genetic variants.

Several pertinent questions remain. Do lipid and lipoprotein processing microglia prevent or promote AD neuropathogenesis? To gain deeper insight, more recent studies have further segregated DAM populations into two ontogenetically and functionally distinct cell populations (32). The first population, bona fide DAMs, resembles developmental microglia and is highly phagocytic and enriched in lipid processing transcripts (e.g., *SPP1*, fatty acid binding protein 5) (32). In contrast, the second population, referred to as disease inflammatory macrophages, appears to be derived from peripheral myeloid cells and exhibits a transcriptional signature indicative of immunosuppression yet inflammation (e.g., interleukin 1 beta, *TNF*, CC chemokine receptor 5, C-C motif ligand 2, and signal transducer and activator of transcription 1) (32). The functional differences between DAMs and disease inflammatory macrophages, namely increased phagocytosis and reduced inflammation, suggest that enhanced lipid metabolism may indeed be beneficial in the context of AD. However, it is important to note that there are differences between DAMs and microglia that accumulate excessive numbers of LDs (24). For example, excessive LD accumulation is associated with reduced phagocytosis (24), which could further exacerbate amyloid load and neurodegeneration in AD. Indeed, emerging studies targeting LD accumulation in microglia have shown improved cellular functions and reduced AD pathology (33). Since LDs are critical cellular organelles, it is likely that the relationship between microglial LD accumulation and functionality is not linear and warrants further study. In addition, since LDs sequester intracellular lipids and protect against lipid peroxidation, strategies that aggressively seek to prevent LD formation should be approached with caution. In light of this, it is essential to investigate the processes upstream of LD accumulation, such as intracellular and intercellular lipid and lipoprotein transport, to identify alternative targets that prevent unwanted lipid accumulation without compromising lipid sequestration.

## Do Microglia Contribute to Brain Lipid Composition and Metabolism?

Lipids are critical to CNS function, serving as structural elements in cell membranes, signaling molecules, and energy storage mediums. The brain is the second most lipid-rich organ in the body after adipose tissue and relies heavily on lipids for optimal function. Lipid metabolism in the CNS is a complex process involving synthesis, transport, remodeling, and catabolism, which are all tightly regulated to ensure proper neuronal and glial function. The primary lipid classes in the brain are cholesterol, PLs, and sphingolipids, with neutral lipids, such as CEs and TGs, being present to a lesser extent in the typical brain. Understanding changes in brain lipid composition with aging and AD has helped identify metabolic processes that could be targeted to improve disease outcomes. However, historically, brain lipid composition has been viewed through a macroscope lens. Exciting recent studies have highlighted region- and cell-specific lipid composition, raising the question: are changes in microglia abundance and function contributing to changes in brain lipid composition in aging and AD?

### Phospholipids

PLs are the most abundant class, accounting for upward of 55% of the total lipid and consist primarily of PCs and phosphatidylethanolamines (PEs) and, to a lesser extent, phosphatidylserines (PSs) and phosphatidylinositols (PIs) (34, 35). PLs are integral to cell membrane structure, impacting membrane fluidity and enabling signal transduction. Their synthesis occurs in both neurons and astrocytes, predominantly within the endoplasmic reticulum. The types of PLs produced influence neurotransmitter release, receptor function, and neuronal-glial communication. PL levels are so precisely coordinated in the brain that small changes in PL levels and accessibility initiate changes in neuronal-glial signaling. For example, microglia are able to sense apoptotic neurons due to the exposure of PS on the cell surface, which acts as an “eat-me” signal and promotes synaptic pruning (36). Hence, PL composition is tightly regulated in the brain in a spatial, temporal, and cell type-specific manner.

PL composition has been shown to generally decrease with aging (37), particularly PLs and PEs containing arachidonic acid (ARA, 20:4) and adrenic acid (derivative of 22:4) (37, 38). Decreased PI, PE, and ethanolamine plasmalogens have been reported in the frontal, temporal, and parietal cortex of AD brains (39, 40, 41). While studies in rodent models of AD have shown that PL supplementation can improve cognition (42, 43), similar improvements have not yet been validated in human studies, which may relate to the complexity of PL composition. Indeed, PS containing steric (C18:0) and DHA (C22:6) are markedly increased (approximately one-third) in the mitochondrial and microsomal membranes of the human prefrontal cortex (38). The increase in this specific PS suggests that the FA composition of PLs is just as important as the PL species. Interestingly, treating microglial cells (N9) with DHA leads to an attenuated response to inflammatory stimuli, such as lipopolysaccharides (LPSs), an increase in microglial PS, reduced LD size, and promotes the interaction between microglial LDs and mitochondria (44). It remains to be tested whether changes in DHA-containing PSs in the whole brain are driven by lipid remodeling in microglia in vivo. However, an increased abundance of long-chain PUFAs (LC-PUFAs), such as ARA, docosatetraenoic acid (C22:4), and DHA has been observed in microglia derived from murine models (App^NL-GF^) of AD (45). In support, ARA-containing PC is also upregulated in microglia of murine models of amyloidosis (amyloid precursor protein [APP]-KI) (33). This is perhaps unsurprising given that LC-PUFAs are precursors to inflammatory modulators readily made by microglia, such as eicosanoids, which may be generated in a “less controlled” fashion in AD, contributing to neuroinflammation. Therefore, it is plausible to suggest that specific PUFA-containing PLs are more or less abundant in the aging and diseased brain due to the inflammatory profile of microglia and their concomitant microglial lipid composition. Further studies are warranted to determine whether changes in microglial number can at least partially account for altered PLs in normal aging.

LC-PUFAs are sequestered as fatty acyl side chains of PLs at cell membranes. Membrane PLs are fluidic and responsive to the changing bioenergetic needs of the cells. Up to 50% of membrane PLs are PC (46), with the synthesis and breakdown of PC being referred to as the Lands cycle (47). Within the Lands cycle, lysophosphatidylcholine acyltransferase combines lysophosphatidylcholine (LPC) with acyl-CoAs, generating PC that can be incorporated into the cell membrane. Reciprocally, FA side chains in the *sn*-2 position of PC can then be mobilized via the action of phospholipase A2, contributing to the pool of LPC (48). Microglia predominantly express lysophosphatidylcholine acyltransferase 3, which preferentially introduces LC-PUFAs onto the *sn*-2 position of LPC (49). Interestingly, genetically depleting *LPCAT3* from microglia of App^NL-GF^ mice has been reported to promote microglial phagocytosis, facilitate de novo lipogenesis, protect microglia from oxidative damage, and lead to a compensatory increase in MUFAs (45). Such an improvement in microglial function following *LPCAT3* depletion is intriguing, since *LPCAT3* depletion would presumably promote the intracellular pool of LPC, which has been linked to the inflammatory polarization of microglia (50, 51). However, recent studies have highlighted the diverse and bioactive nature of lyso-PLs (52) and suggest that circulating LPC may provide the LC-PUFAs that are otherwise depleted in the aged brain (53). In addition, it is plausible to suggest that in microglia at later AD stages, metabolic triggers may actually prevent microglial exhaustion and promote relatively adaptive cellular functions in the diseased brain. Further work is needed to ascertain whether PL delivery and remodeling within microglia is a rational target for the treatment of AD.

### Cholesterols

Compared with other organs and tissues, the brain is remarkably cholesterol-rich. This is likely because cholesterol is synthesized de novo in the CNS, predominantly by astrocytes, neurons, and mature oligodendrocytes. The latter of which are responsible for the synthesis of myelin, which is 40% cholesterol (54, 55). As well as myelin formation, cholesterol is important for maintaining membrane integrity, facilitating synaptic transmission in neurons, and providing trophic support to other glial cells, which synthesize cholesterol at much lower levels, that is, microglia.

**Fig. 2:**
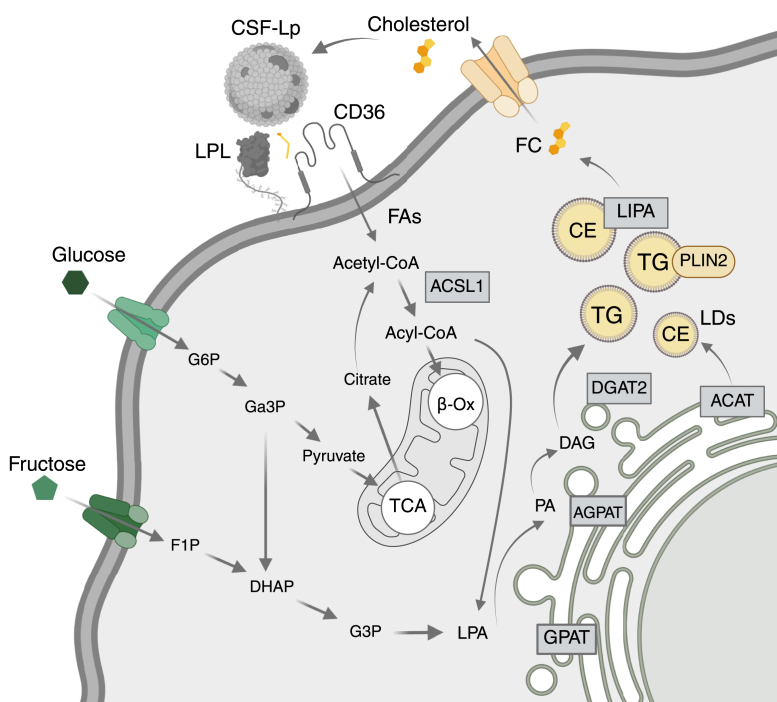
**De novo lipogenesis in microglia.** FAs enter the cell *via* LPL-mediated hydrolysis and CD36-mediated FA transport, contributing to the intracellular pool of acetyl-CoA. ACSL1 converts acetyl-CoA to acyl-CoA, which can then undergo oxidation in the mitochondria or contribute to de novo lipogenesis by feeding into the pool of LPA. In the ER, LPA gets converted into PA, DAGs, and eventually TAGs, the rate-limiting enzyme being DGAT2. Cholesterol is esterified by ACAT. CE and TAGs are then stored as LDs. Glycolytic pathways, such as glycolysis and fermentation, also contribute to de novo lipogenesis by contributing to the pools of G3P and LPA. ACAT, acyl-CoA:cholesterol acyltransferase; ACSL1, acyl-CoA synthetase long-chain family member 1; CD36, cluster of differentiation 36; DAG, diacylglycerol; DAGT2, diacylglycerol O-acyltransferase 2; DHAP, dihydroxyacetone phosphate; F1p, fructose 1-phosphate; FA, fatty acids; G3P, glyceraldehyde-3-phosphate/Ga3P; GPAT, G3P acyltransferase; LD, lipid droplet; LPA, lysophosphatidic acid; LPL, lipoprotein lipase; PA, phosphatidic acid; TAG, triacylglycerol.

CE represents a means of cholesterol storage within cells, helping manage free cholesterol levels and preventing toxicity. The formation of CEs involves the esterification of cholesterol with FAs catalyzed by ACAT, predominantly occurring in the endoplasmic reticulum. These esters can be stored in LDs and mobilized when necessary, ensuring a balance between free and esterified cholesterol within cellular compartments. For cholesterol to be mobilized and effluxed from the cell, CEs within LDs are subject to de-esterification by lysosomal acid lipase, encoded by *LIPA*. The resulting free cholesterol can then be used for membrane and lipid raft formation, reparative processes, or effluxed and incorporated into lipoproteins for lipid recycling (Fig. 2). In microglia, there has been considerable interest in CE accumulation within LDs, which are associated with impaired cellular functions and even cellular senescence. CE accumulation has also been reported in the AD brain and in ApoE4-carrying astrocytes (56). Considering *LIPA* is predominantly expressed by microglia of the murine and human brain, and has the capacity to mobilize cholesterol from LDs, it is surprising *LIPA* has not yet been pursued as a target for improving microglial function in AD.

Another fate of free cholesterol is the conversion into oxysterols such as 25-hydroxycholesterol (25HC), which is synthesized by cholesterol-25-hydroxylase (CH25H), encoded by the *CH25H* gene. 25HC has received considerable interest considering its ability to metabolically reprogram immune cells (57). Conversely, elevated 25HC has been observed in models of infection and inflammation (58). Recent studies have also shown that *CH25H* is overexpressed in the human AD brain and in mouse models of amyloidosis and tauopathy (59). In fact, *CH25H* appears to be a feature of DAMs but only in the presence of TREM2 and ApoE (18, 60). Mechanistically, *CH25H* is thought to be induced by the Toll-like receptor 4 (TLR4) agonist, LPS (61). In primary mouse microglia, LPS treatment leads to elevated *CH25H* expression and 25HC secretion. Notably, enhanced 25HC secretion is exacerbated in ApoE4-carrying microglia, compared with ApoE2 or ApoE3, supporting the prevailing hypothesis that ApoE4-expressing microglia exhibit a more classically inflamed phenotype. Since 25HC is secreted, Cashikar *et al.* hypothesized that 25HC may act as a signaling molecule involved in regulating cholesterol homeostasis in the CNS more broadly. Supplementing astrocytes with 25HC resulted in increased astrocytic ApoE-containing lipoprotein production, without an increase in ApoE mRNA or cholesterol synthesis, suggesting that the efflux of poised ApoE was primarily involved (62). Notably, 25HC-mediated cholesterol efflux was more pronounced in ApoE3-carrying cells than ApoE4. Interestingly, 25HC also doubled the quantity of CE in astrocytes, leading to enhanced astrocytic LD formation (62). Since increased ApoE and CE formation have been repeatedly linked to AD, it is reasonable to suggest that microglial 25HC may be a maladaptive response to inflammatory insults that promote AD neuropathogenesis. Indeed, in a recent study, genetic depletion of *CH25H* in a rodent model of tauopathy was sufficient to reduce phosphorylated tau but did not prevent tau seeding or spreading (63). Microglial phenotype also appeared to be shifted toward a more homeostatic state following *CH25H* depletion, with an increase in homeostatic markers, such as transmembrane receptor 119 (Tmem119) and purinergic receptor P2Y12 (P2Ry12). Future studies are needed to determine whether pharmacological manipulation of *CH25H* and resulting 25HC levels are viable targets to improve the function of AD-susceptible microglia to improve AD outcomes (63).

However, similar to targeting LD accumulation, targeting cholesterol synthesis and metabolism within the CNS and microglia is not a simple task, and whole-body cholesterol homeostasis needs to be considered. In the circulation, the LDL-C increases until adulthood, but then declines in later life, which involves both reduced dietary absorption and hepatic synthesis (64). Therefore, the fact that in aged individuals, low LDL-C is associated with poor health outcomes and increased mortality (65), and aged individuals may be at an increased risk of developing side effects from cholesterol-lowering medications, is perhaps not as controversial as it initially seems (66). Indeed, declining cholesterol levels have also been reported in the aging brain. It has been reported that in typical brain aging, cholesterol levels fall by around 50%. In addition, the fall is more pronounced in myelin lipids, especially in females over 70 years old (37), which may contribute to the increased risk of age-associated neurodegenerative disease in women. In addition to the reduced cholesterol content in myelin with age, the rate of myelin recycling and repair is also thought to be reduced, largely through poor recycling of cholesterol from CNS microglia (67). Indeed, cholesterol transport by ApoE-containing lipoproteins is thought to be impaired in the presence of ApoE4, the strongest genetic driver of AD (56, 68). Despite impaired transport of cholesterol, recent studies have shown that in human microglia and astrocytes, ApoE4 expression is associated with impaired cholesterol trafficking, reduced cholesterol efflux, yet increased de novo lipogenesis of cholesterol in astrocytes (69). It is plausible that the increased de novo synthesis is a compensatory response to the impaired cholesterol recycling. Taken together, these studies highlight the need for interventions that improve cellular transport of cholesterol rather than simply modifying cholesterol abundance. To facilitate the development of such interventions, it is necessary to carefully consider the mechanisms governing cholesterol transport between microglia, other glial cells, and neurons in the typical, aging, and diseased brain (69).

### Sphingolipids

The CNS is rich in sphingolipids, which play a key role in lipid signaling, cell and membrane biology, and inflammation, but a lesser role as an energy source compared with other brain lipids. Sphingolipid metabolism is a complex network of metabolic pathways that seem to converge with the generation of a relatively simple sphingolipid, ceramide (Cer), which can serve as an intermediate to synthesize more complex sphingolipids such as SM and glycosphingolipids (for a comprehensive recent review on brain sphingolipids, see Ref. (70)). Several studies have also shown that circulating sphingolipid metabolism is robustly dysregulated in individuals with mild cognitive impairment in the early stages of AD, highlighting its utility as an early indicator of AD neuropathogenesis (71, 72). In the brain, SMs are primarily found in lipid rafts of cell membranes and play important roles in signal transduction, inflammation, and the response to oxidative stress (73). In typical brain aging, and in the AD brain, the abundance of Cers increases, particularly long-chain (C24) Cer species (74). Interestingly, recent studies using induced pluripotent stem cell (iPSC)-derived cells have shown that neurons accumulate C16 and C18 Cers, astrocytes accumulate C24:0 Cers, but microglia have the highest C24:1 content. In addition, comparative transcriptomic analysis of different cell types showed that microglia have higher relative expression of the genes involved in Cer synthesis, such as Cer synthase 1, and Cer conversion, such as acid ceramidase and alkaline ceramidase 3, suggesting that microglia are capable of de novo Cer synthesis and conversion to sphingosine (75). These findings have been corroborated by recent cell-specific lipidomic analyses showing that Cer and SM are particularly enriched in microglia compared with other CNS-resident cells (6). Taken together, these findings highlight the need to determine whether changes in microglial abundance and function contribute to the accumulation of long-chain Cer in the aging and AD brain.

In addition, there is a need to understand the consequences of elevated, microglia-derived Cer synthesis in the aging and diseased brain. Recent studies have demonstrated that experimentally inducing Cer accumulation by supplementing with a glucosylceramide synthase inhibitor results in marked upregulation of inflammatory transcripts in microglial cells (75). The notion that Cer accumulation is linked to inflammatory polarization of microglia is supported by recent studies investigating the role of Cer-rich extracellular vesicles (EVs) in AD pathology (76). Characterization of microglia-derived EVs from AD brains has revealed enrichment in monohexosylceramides, notably monohexosylceramide 18:1/24:1, consistent with the in vivo data outlined above reporting synthesis of C24:1 Cer in microglia. These robust findings highlight the utility of EV isolation and characterization as a biomarker for AD onset and progression. Given the emerging role of microglia-derived EVs in the transport and neurotoxic spread of the Aβ (77, 78), it is tempting to ask whether halting microglia-derived EVs transport is neuroprotective (77, 78). Indeed, recent studies have shown that preventing the movement of microglia-derived EVs carrying Aβ reverses synaptic dysfunction in entorhinal-hippocampal neurons (79).

As previously outlined, Cer can be converted to sphingosine, which is in turn phosphorylated to sphingosine-1-phosphate (S1P) by sphingosine kinases. Notably, the brain has the highest S1P context, which may relate to the fact that microglia, oligodendrocytes, and neurons abundantly express S1P receptors. It has been reported that microglia express S1P receptor 2, and that in response to neuronal S1P accumulation, microglia become de-ramified and proinflammatory in vivo and in vitro (80). Notably, increased signaling through this pathway has been noted in 5xFAD mice but can be at least partially resolved with the sphingosine-1-phosphate receptors 1 and 2 inhibitor fingolimod (81). In further support, the sphingosine-1-phosphate receptor 1 antagonist, ponesimod, prevents Aβ-induced activation of microglia, to reduce neuroinflammation and increase Aβ clearance in vivo (82). Taken together, this highlights a mechanism by which sphingolipids regulate neuron-microglia crosstalk and highlights the therapeutic potential of inhibiting SIP signaling in AD. However, since S1P is carried by apolipoprotein M in the periphery (83), future studies are needed to address whether S1P is also carried in BLps, and which apolipoproteins it favors. This is particularly pertinent, given the sometimes-muddy distinction between microglia-derived EVs and BLps.

### Triglycerides

TGs, while more prominent as energy stores in peripheral tissues, also play a key role in the CNS. They are synthesized through the esterification of three FA molecules with glycerol-3-phosphate, in a process facilitated by diacylglycerol (DAG) O-acyltransferase (DGAT). TGs are stored in LDs within cells and can be hydrolyzed to release FAs, which serve as substrates for energy production through β-oxidation during periods of high metabolic demand or stress. Recent lipidomic analysis of the brain has shown that TGs are abundant in regions enriched in white matter (6). In addition, several recent studies have shown that microglia are particularly enriched in TGs (6, 14), and that TG accumulation in microglia increases in response to factors that promote AD pathology, such as murine and cell models of ApoE4 expression (14), and murine models of amyloidosis (e.g., APP-KI) mice (33). The fact that TG accumulation increases in the AD brain and with age prompts the question of whether microglial cell expansion, or even infiltration of myeloid-derived cells that are also rich in TG, contributes to increased composition.

Why microglia preferentially accumulate TGs is a driving question in the field. Typically, increased TGs result from excessive lipid supply. However, increased TGs and subsequent LD accumulation are also a product of reduced lipid supply, and an immunometabolic shift toward carbohydrate utilization and glycolysis, which in turn drives de novo lipogenesis. It is therefore notable that in response to inflammatory stimuli such as Aβ, microglia markedly upregulate LD accumulation, robustly in vivo and in vitro (14, 31, 84). Several groups have independently shown that LD accumulation in microglia is dependent on enzymes in the TG synthesis pathway. For example, when comparing the transcriptional signature of microglia isolated from control brains versus individuals with AD who are homozygous for *APOE4*, Haney *et al.* found that the most differentially expressed gene was acyl-CoA synthetase long-chain family member 1, which converts long-chain FAs into the fatty acyl-CoAs, preferably C18:1 and C18:2 (85), and one of the first steps in the synthesis of TGs (Fig. 2). Single-nuclei RNA sequencing also highlighted an ACSL1+ microglial subtype that coexpressed genes associated with lipid synthesis and colocalized with amyloid plaques. In vitro, analysis combined with label-free lipid imaging (coherent anti-Stokes Raman scattering) showed that Aβ stimulation could increase ACSL1+ expression and TG accumulation. Importantly, treatment with the ACSL1 inhibitor, Triacin C, reversed the Aβ-dependent accumulation of LDs in ApoE4 microglia. While this suggests that ACSL1 may be a rational target to improve microglial lipid metabolism and hence function in the AD brain, it is important to consider that ACSL1 is involved in more than just TG synthesis in the cell. For example, the conversion of FAs to acyl-CoAs is also a rate-limiting step in the partitioning of FAs toward β-oxidation. Indeed, transgenic mice lacking ACSL1 exhibited 50–80% reduction in FA oxidation and a 30% greater fat mass, even on a normal diet (86). Therefore, it is tempting to speculate that an upregulation of ACSL1 may, in fact, be an attempt to increase energy production from alternative energy sources (e.g., FAs) in microglia that are under considerable metabolic stress and trying to resolve Aβ deposition and other facets of AD neuropathogenesis. Nonetheless, during such inflammatory conditions, there is often a concomitant increase in glucose and fructose uptake and hence increased glycolytic intermediates that shunt metabolic flux (22), and presumably *ACSL1* activity, toward TG synthesis and away from FA oxidation (Fig. 2). This is also consistent with the prevailing hypothesis that ApoE4 is poorly lipidated and leads to poor lipid supply and metabolic switching to glycolysis (87). Further studies that address metabolic flux in microglia will be particularly informative in deciphering the interaction between ApoE4 and TG synthesis. In addition, strategies that modify the uptake and breakdown of sugars should be considered alongside strategies aimed at lowering TGs.

Further support for increased TG synthesis leading to microglia dysfunction is provided by observations that DGAT2, which catalyzes the final step in TG synthesis by catalyzing the formation of an ester linkage between a fatty acyl-CoA and the free hydroxyl group of DAG, is upregulated in both human AD brains and microglia isolated from AD-susceptible 5xFAD mice. Importantly, inhibition or degradation of DGAT2 resulted in improved microglial phagocytosis of Aβ and led to reduced Aβ load in 5xFAD mice (84). Taken together, these findings highlight the need for further studies that determine whether DGAT2 is a viable target to improve LD formation and microglial function in AD. Since the primary substrate for DGAT2 activity is DAG, the pool of which is increased following increased shunting of glycolysis and fructolysis intermediates through the glycerol-3-phosphate-lysophosphatidic acid-phosphatidic acid-DAG pathway (Fig. 2), future studies that consider the flux of synergistic metabolic pathways are also warranted.

It is important to consider how microglia gain access to substrates that promote TG accumulation, and therefore, circulating lipids cannot be overlooked entirely. In the circulation, TGs are transported in TG-rich lipoproteins (TRLs), which cannot cross the blood-brain barrier (BBB) under typical conditions. However, whether TRL transport occurs during inflammation, disease, or damage to the brain cannot be entirely ruled out. Interestingly, increased systemic TGs have been linked to both an increased and decreased risk of AD. Increased TRLs, particularly large and medium LDLs, have been reported to be strongly associated with AD progression and Aβ deposition (88). Intermediate density lipoproteins and VLDLs, which are typically very TG-rich, were not associated with Aβ deposition (88). In contrast, large HDLs were associated with a decreased risk of Aβ pathology (88). These findings suggest that TGs per se may not drive AD risk, but rather the lipoprotein composition or the functionality of factors that regulate lipoprotein metabolism. Indeed, this notion is supported by a recent study showing that older individuals with TG levels within the normal to high-normal range had a lower dementia risk and slower cognitive decline compared with individuals with relatively low TGs. This suggests that reduced circulating lipids may be detrimental to overall brain health and warrants further studies that define which specific lipoprotein components offer a specific benefit in AD and how these circulating lipids access the brain to influence brain lipid composition and metabolism.

## How do Microglia Access and Use Lipids?

The delivery of lipids within the CNS is accomplished through lipid-protein complexes known as lipoproteins, which navigate the aqueous extracellular space to transfer lipids across brain regions and between cell types. In the peripheral circulation, lipoproteins are typically classified based on particle density into TG-rich VLDLs, cholesterol-rich LDLs, and protein-rich HDLs. Lipoproteins are mostly spherical and feature a neutral lipid core composed of CEs and TGs, encapsulated by a PL monolayer containing free cholesterol. Apolipoproteins serve as dynamic organizing scaffolds that solubilize the lipids for transport through the aqueous environment and serve as ligands for a variety of lipoprotein receptors on target cells. While the transport of lipoproteins in the circulation is rather well established (for a comprehensive recent review of lipid and lipoprotein metabolism, see Ref. (89)), our understanding of lipoprotein metabolism in the CNS is poorly understood and remains an active area of study. Understanding the basic mechanisms underlying lipoprotein transport in the brain will improve our understanding of lipid supply to microglia and will greatly inform our understanding of AD pathogenesis, given that variants in key apolipoproteins (e.g., ApoE4) and microglia-specific lipoprotein receptors (e.g., TREM2^R47H^) are the strongest genetic drivers of AD.

### Cerebrospinal fluid-lipoproteins (CSF-Lps)

The CNS relies on horizontal lipid flux between neurons and glial cells to maintain homeostasis, and lipid delivery is facilitated by lipoproteins in the CNS. These lipoproteins are thought to be generated mostly de novo in the CNS and primarily reside in the cerebrospinal fluid (CSF), where they exist at much lower concentrations than their plasma counterparts. Indeed, based on measurements of cholesterol and PLs, the concentrations of lipoproteins in CSF-lipoproteins (CSF-Lps) are approximately 350 times lower than those of plasma-Lps (90). Despite their low abundance, studies have revealed that CSF-Lps are essential for the formation and maintenance of neuronal membranes and myelin sheaths (91), underscoring the importance of lipid crosstalk between cells to ensure the metabolic requirements of neurons are met to maintain functional synaptic signaling and plasticity.

Due to their low abundance, as discussed above, the detailed biochemical characterization of CSF-Lps has been historically limited. Nonetheless, cursory biochemical analysis of CSF-Lps has reported the particles deficient in ApoB100 that define TRLs, such as chylomicrons, VLDL, and LDL (92) and enriched in apolipoproteins, such as ApoA1 and ApoE, suggesting they most closely resemble plasma HDL. While best recognized for its ability to mediate cholesterol efflux from artery walls, HDL is also now appreciated to play roles in modulating systemic immune responses (93), inflammation (94), glucose metabolism (95), and oxidative stress (96)—functions that are critical for healthy metabolism in the CNS, and when dysfunctional, are linked to neurological disease development. Recent technologies, such as fluorescent lipoprotein profiling coupled with ultrasensitive proteomics, have revealed that HDL-like, CSF-Lps are a highly heterogeneous population of particles (16). However, while HDL-sized populations exist, the predominant CSF-Lp species are slightly larger than traditional plasma HDL. Proteomics and lipidomics analyses have revealed that CSF-Lps exhibit incredible compositional diversity, containing over 300 proteins (16) and 200 lipids. This suggests that, like plasma HDL (97), CSF-Lps contain compositionally distinct particle subspecies that have unique molecular signatures and potentially distinct downstream effector functions (for a comprehensive recent review, see Ref. (98)).

### Apolipoprotein E (ApoE)

Even across diverse subspecies, CSF-Lps contain an abundance of ApoE. ApoE is synthesized de novo entirely within the CNS and does not appear to cross the BBB (99). Human ApoE is polymorphic, resulting in several variant ApoE proteins. The three main isoforms of ApoE are ApoE2, ApoE3, and ApoE4. It is well documented that ApoE4 is the strongest genetic driver of late-onset AD and is present in 55–75% of AD dementia cases (100). In the brain, ApoE is involved in a variety of critical functions, such as lipid and cholesterol transport between neurons and glial cells, immunometabolic regulation, and synaptogenesis (87, 98, 101). Recent studies have also highlighted a novel role of ApoE, as an LD-associated protein that regulates both LD biogenesis and composition in astrocytes (102, 103). Notably, in this role, ApoE4 acts as a toxic hypermorph, leading to larger LDs with poor turnover, which may at least in part explain the impaired lipid transport associated with ApoE4 (103). This is consistent with the fact that astrocytes produce most of the ApoE in the CNS, which is thought to be up to 80% of the total ApoE in the murine brain (104). Therefore, defects in astrocytic cholesterol processing likely impact horizontal lipid flux between astrocytes, microglia, neurons, and other cells in the brain. This crosstalk is likely bidirectional, a notion that is supported by the fact that microglia-derived 25HC leads to a 2-fold increase in astrocytic ApoE production (105). Although increased 25HC and ApoE are associated with AD neuropathogenesis in human AD brains, murine models of AD, and ex vivo studies, it remains to be determined whether this is actually an attempt for the brain to increase lipid transport to initiate reparative processes. In addition, the effect of astrocyte-derived ApoE on microglia is an active area of research. The majority of studies have focused on immunometabolic changes to microglia that occur following ApoE4 expression, which has been robustly linked to LD accumulation, proinflammatory immunometabolic polarization, and cellular dysfunction in murine models of neurodegeneration (106, 107). However, the effect of astrocyte-derived ApoE-containing lipoprotein on microglial metabolism, function, and influence on disease state has yet to be empirically defined. Further studies are also required to determine the effect of the ApoE lipidation status on lipid delivery to microglia and consequent immunometabolic polarization of the cell.

### Apolipoprotein A1 (**ApoA1**)

ApoA1 is imported from the periphery through the BBB or blood CSF barrier (108). The pathway of ApoA1—whether as a poorly lipidated protein or as a small HDL particle traversing these barriers—remains under investigation. Recent studies have shown that ApoA1 is absent from the CSF of intestine and liver-specific *ApoA1* KO mice, showing that at least in mice, ApoA1 present in the brain is derived from the intestine and liver (109). Consequently, CSF-Lps may constitute a mix of BLps and peripheral lipoproteins entering the CNS, both contributing to lipid dynamics in the CSF. Although it is likely that the type of BLps generated de novo by astrocytes results from lipid-free ApoE and ApoA1 interacting with ABCA1 at the cell surface, facilitating cholesterol efflux and forming initial discoidal particles akin to HDL in plasma, studies empirically defining this process are lacking. Within the CSF, lecithin-cholesterol acyltransferase can interact with both lipid-bound ApoA1 and ApoE to convert free cholesterol into CEs, fostering the maturation of spherical or discoidal lipoprotein particles with a neutral lipid core (109).

ApoA1 promotes the efflux of cholesterol and PLs through the interaction with ABCA1 (110). Given its potency as a cholesterol acceptor, ApoA1 mimetic peptides have been considered as a therapeutic strategy to reduce foam cell formation in atherosclerosis (111). Since lipid-laden microglia overlap phenotypically with macrophage foam cells, it is plausible to suggest that ApoA1, or indeed ApoA1, mimetics may promote cholesterol efflux from microglia to improve cellular functions such as phagocytosis. Indeed, in a recent study, an ApoA1 peptide mimetic (5A) was shown to increase LD accumulation in microglia in response to myelin, suggesting increased uptake and reutilization of myelin-derived lipids. In support, 5A can improve remyelination in vivo but not after microglial depletion (112). Mechanistically, 5A is thought to increase the uptake of myelin-derived lipids by increasing the expression of the FA transporter cluster of differentiation 36 (112). While these studies support the idea that ApoA1 can be detected by microglia and modify microglial lipid processing, the mechanisms of microglial-ApoA1 sensing are only recently being defined. An intriguing recent study assessing BBB penetrance has shown that hypothalamic microglia readily engulf plasma ApoA1, which in turn modulates microglial function (113). Specifically, ApoA1 exposure can attenuate the inflammatory response to LPS by microglia and can improve phagocytosis of Aβ. Although this process may be more relevant in privileged sites of the brain, with increased access to the plasma proteome, this raises questions of whether ApoA1 is a viable therapeutic to improve microglia function. Indeed, to develop such a strategy, the mechanisms by which ApoA1 interacts with microglia require further study.

Some potential insights surrounding the mechanisms of microglia ApoA1 sensing can be extrapolated from circulating lipoproteins and macrophages. In the periphery, *ApoA1*-containing lipoproteins are thought to preferentially interact with scavenger receptor class B type 1 (SR-B1), delivering lipid cargo to SR-B1-expressing target cells and being cardioprotective by inhibiting the binding of LDL to SR-B1 (114). Notably, SR-B1 is expressed in the brain and abundantly expressed in microglia and brain macrophages (115), where it binds to fibrillar Aβ. Conversely, depletion of SR-B1 leads to impaired perivascular macrophage (PVM) function and enhanced Aβ deposition (116). Therefore, it is reasonable to hypothesize that *ApoA1*-containing lipoproteins may interact with microglial SR-B1, which would in turn improve microglial lipid metabolism and AD risk. Moreover, given the fact that increased plasma levels of ApoA1 are associated with improved cognitive performance (117), it is surprising that the neuroprotective effects of ApoA1 and its actions on microglia have been somewhat overlooked.

### Secreted phosphoprotein 1 (SPP1)

It has been demonstrated that CSF-Lps are heterogeneous, containing diverse proteins with distinct roles (16). Given that astrocytes are the primary source of BLps, their enrichment in neuron-supportive proteins implies that CSF-Lps facilitate crosstalk within the CNS. Intriguingly, distinct clusters of CSF-Lps defined by SPP1 have been identified (16), which has been linked to activated macrophages and microglia. AD-associated microglial subpopulations, in both human and mice, are robustly defined by upregulated expression of lipoprotein components and regulating factors, such as *ApoE*, *LPL*, *TREM2*, and *SPP1* expression (118), suggesting microglial lipoprotein production is a critical response to AD pathology. Recent studies have shown that SPP1 is predominantly expressed by PVMs, as well as in human AD tissues, where it appears to be upregulated following microglial synapse elimination. Conversely, microglial-synapse phagocytosis is reduced following genetic ablation of *SPP1*, suggesting that SPP1 presents an extrinsic signal that mediates crosstalk between PVMs, microglia, and neurons in the AD brain (119). Despite the clear role of SPP1 in microglial function and AD, the mechanisms by which SPP1 is carried in CSF-Lps and detected by target cells remain to be determined.

In summary, throughout their lifespan, lipoproteins in the CNS interact with a plethora of enzymes and cell surface receptors thought to remodel CSF-Lps and contribute to their compositional heterogeneity. Structural regulation by scaffolding proteins like ApoA1, with its ability to adopt various conformations (120), suggests an adaptive mechanism for facilitating these interactions in the extracellular space and with various receptors at the cell surface. ApoE dynamics on the lipoprotein surface, while potentially analogous, are less understood. Overall, further investigation into CSF-Lps, their receptors, and their roles in lipid delivery and modulation of cell function is warranted, particularly concerning their impact on microglial dysregulation in AD development and progression. Further studies are also needed to determine whether there is a differential ratio of CSF-Lps and peripheral particles in disease and how this ratio may change throughout aging and disease. An important question that requires clarification is whether CSF-Lp composition is altered in AD and whether structural defects of scaffold proteins impact this composition.

## Lipoprotein Receptors: Immunometabolic Gatekeepers of Microglial Metabolism and Function?

### Triggering receptor expressed on myeloid cells 2 (TREM2)

**Fig. 3:**
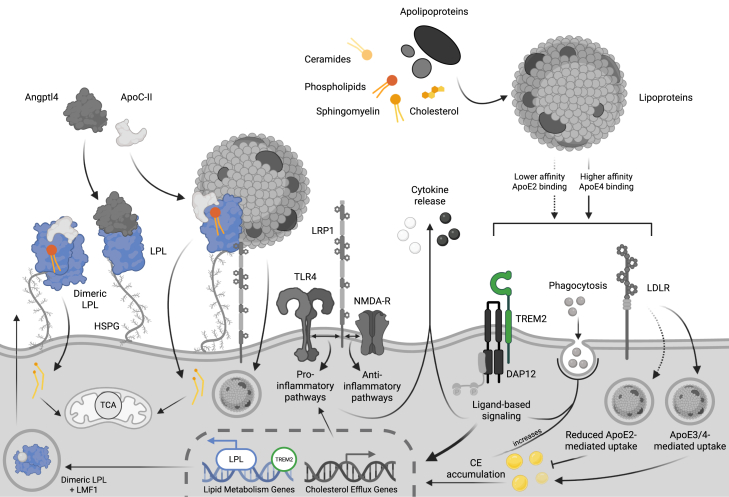
**Receptor-mediated pathways in microglia. Apolipoproteins and lipids combine to form lipoproteins, which bind to microglial receptors. Lipoproteins are endocytosed by LRP-1 and LDLR, and their components are used as structural components and potentially metabolic substrates.** ApoE-containing lipoproteins differentially bind to lipids and receptors, with reduced binding and uptake correlated with ApoE2, and increased with ApoE4. Differences in internalized lipid cargo affect CE accumulation, driving transcriptional changes in cholesterol efflux genes and phagocytosis. These processes are further influenced by ligand-based signaling, from complexes like TREM2-DAP12. Signaling and receptor-receptor interactions, like TLR4 or NMDA-R with LRP-1, ultimately drive anti-inflammatory and proinflammatory pathways that produce cytokines. Transcriptional shifts also upregulate lipid metabolism genes like LPL, which is expressed and tethered to the membrane via HSPGs. LPL activity and function is modified by cofactors like ApoC2 and Angptl4. Angptl4, angiopoietin-like 4; ApoC-II, apolipoprotein C-II; ApoE, apolipoprotein E; CE, cholesterol ester; DAP12, DNAX-activating protein 12; HSPG, heparan sulfate proteoglycan; LDLR, low-density lipoprotein receptor; LMF1, lipase maturation factor 1; LPL, lipoprotein lipase; LRP1, low-density lipoprotein receptor-related protein 1; NMDA-R, N-methyl-D-aspartate receptor; TCA, tricarboxylic acid cycle; TLR4, toll-like receptor 4; TREM2, triggering receptor expressed on myeloid cells 2.

Microglial metabolism is intricately regulated by receptor-mediated lipid uptake, a process governed by a suite of lipoprotein receptors that orchestrate lipid trafficking, immune signaling, and phagocytic activity (Fig. 3). Among these, TREM2 has garnered substantial attention, yet findings remain inconsistent and context-dependent. In myelin-treated TREM2-deficient murine macrophages and human iPSC-derived microglia, the loss of TREM2 led to impaired myelin cholesterol clearance and accumulation of CEs, despite intact phagocytosis of myelin debris (26). This indicates that TREM2 is required for the phagocytosis of lipid-rich debris, such as myelin, and suggests that this role is conserved across species. Similarly, across in vitro, coculture, and in vivo models of ischemic stress, TREM2 deficiency led to LD formation, suppression of cholesterol efflux, and a shift toward a proinflammatory, neurotoxic microglial phenotype (121). However, other studies suggest that TREM2 deficiency does not uniformly drive LD accumulation, and that its impact may vary depending on disease context or TREM2 variant. For example, in a humanized chimeric model of AD, Claes *et al.* found that both WT TREM2 and microglia carrying the TREM2 loss-of-function, AD-risk mutation TREM2^R47H^, exhibited foam cell-like transcriptional profiles; however, plaque-associated WT cells were markedly enriched in LDs, whereas TREM2^R47H^ microglia accumulated *fewer* LDs, diminished plaque engagement, and lower ApoE secretion (31). Similarly, Filipello *et al.* demonstrated that iPSC-derived microglia harboring the TREM2 p.Q33X loss-of-function mutation, found in patients with Nasu-Hakola disease, exhibited reduced LD content, downregulation of cholesterol genes, and impaired lysosomal function, emphasizing the mutation-specific and context-dependent effects of TREM2 dysfunction on microglial lipid metabolism (122). These findings are consistent with recent studies suggesting that increased lipid processing, which could be visualized by moderate LD formation, may be beneficial in the context of AD (32).

Recent integrative metabolomic and transcriptomic analyses in APP/presenilin-1 models have further demonstrated that TREM2 deficiency broadly disrupts lipid and sphingolipid metabolism, in part through modulation of LPL and other lipid regulatory pathways (123). Complementing these findings in human tissue, lipidomic profiling of postmortem brains from AD donors carrying rare TREM2 risk variants revealed exacerbated dysregulation of Cer, PS, and SMs compared with AD donors without TREM2 variants (124). Several studies have highlighted a general metabolic defect in microglia following disrupted TREM2 signaling, which can be restored through the use of “metabolic boosters” (e.g., cyclocreatinine) (125). In further support, recent work demonstrated that supplementation with tricarboxylic acid cycle metabolites, such as citrate and succinate, ameliorated oxidative phosphorylation deficits, restored lipid content, and improved Aβ phagocytosis in TREM2^R47H^ human iPSC-derived microglia, highlighting the potential for metabolic interventions to rescue microglial function (126). Importantly, recent studies demonstrate that therapeutic activation of TREM2 can also restore lipid and energy metabolism in microglia. In AD models, treatment with a BBB-penetrant TREM2-activating antibody enhanced mitochondrial FA oxidation, promoted cholesterol clearance, reduced LD accumulation, and improved microglial functional states (127). Similarly, boosting TREM2 signaling using biomimetic nanoparticles improved microglial lipid metabolism, enhanced cholesterol efflux via ApoE and ABC transporters, reduced LD accumulation, and promoted cognitive recovery in a model of developmental neurotoxicity (128), further supporting the therapeutic potential of restoring TREM2-mediated lipid handling.

In addition to intrinsic impairments caused by TREM2 variants, recent work highlights how extrinsic receptor interactions can suppress TREM2-mediated clearance of lipid-associated debris. Zhao *et al.* developed a synthetic Aβ-lipid complex (AOB-lipid) to model the lipid-rich, aggregated substrates encountered by microglia in AD. TREM2 binds to AOB-lipid, triggering downstream activation pathways that promote microglial migration and phagocytosis. However, when leukocyte immunoglobulin-like receptor subfamily B member 2 (LILRB2), an inhibitory receptor coexpressed with TREM2, is simultaneously engaged by the same substrate, it potently suppresses TREM2 signaling. Using this AOB-lipid system, the authors demonstrated that LILRB2-mediated inhibition reduces microglial migration and phagocytosis of amyloid-lipid complexes. Notably, therapeutic blockade of LILRB2 with a monoclonal antibody (Ab29) restored TREM2 activation and enhanced microglial plaque clearance in vivo, identifying a novel axis through which microglial lipid handling might be rescued in AD (129).

Together, these studies suggest that TREM2’s role in microglial lipid metabolism is highly context-dependent. The direction of this effect may depend on the type of metabolic challenge (e.g., ischemia vs. amyloidosis), the specific TREM2 variant involved, or differences in lysosomal burden or lipid substrates across systems. In addition, age and sex are emerging as key modulators of TREM2-associated lipid metabolism in microglia, with recent studies showing that female microglia adopt more pronounced DAM-like transcriptional states during aging, including greater induction of TREM2 and other AD-associated genes, alongside elevated expression of lipid regulatory pathways (130, 131, 132). Whether this increase in TREM2, or indeed an increase in lipid-processing DAM, promotes or protects against the development of AD is a key and pertinent unanswered question. Clues may come from other environmental factors that are known to promote AD pathology, such as microglial pathogens and neurotropic viruses (133). Interestingly, the alphaherpes virus HSV-1 has been linked to both AD pathology in human brains (133), and altered microglial function in rodent models (134), which could potentially be attributed to reduced TREM2 expression (135), and otherwise beneficial phagocytic functions of TREM2-expressing microglia. The exact mechanisms by which TREM2 mediates divergent outcomes remain unresolved but may reflect differences in substrate availability, lipid source, ApoE isoform context, or coregulation by downstream metabolic effectors.

The context-dependent nature of TREM2 is likely a product of TREM2’s ability to bind to a variety of extracellular and intracellular binding partners, mediating its effector functions. In microglia, a key binding partner is phospholipase C gamma 2 (PLCγ2), which is predominantly expressed in microglia of the CNS and variants of which have been linked to AD progression (136). Generally, PLCγ2 is involved in signal transduction, cell differentiation, cell proliferation, cell survival, immunological responses, and microglial lipid metabolism (137). Specifically, activated PLCγ2 converts PI 4,5-bisphosphate into the second messengers inositol trisphosphate and DAG (138). Intriguingly, a hypermorphic gain-of-function variant PLCγ2 (S707Y) leads to chronic hyperactivation and dysfunction in human iPSC-derived microglia, characterized by reduced phagocytosis and cytokine release (136). Whereas analysis of microglia derived from transgenic mice carrying another gain-of-function PLCγ2 variant (e.g., P522R), but on the AD susceptible APP/presenilin-1 background, revealed increased metabolic capacity of microglia and increased coverage of amyloid plaques, presumably to mitigate neurodegeneration (138). While these inconsistencies require further study, it is reasonable to suggest that they largely stem from the increased energetic demands of microglia within an AD-like environment. In addition, in induced microglia in vitro, *TREM2* and *PLCG2* depletion led to similar metabolic defects and lipidome remodeling (139). While at baseline, the changes in the lipidome between WT cells and *TREM2* KO and *PLCG2* KO cells are subtle, involving decreased abundance of a limited number of PL species with similar acyl side chains (e.g., PC 36:2, PE 36:2, PG 36:2), SM, and decreased HexCer (d18:1/16:0). However, the lipidomic changes following a myelin challenge were much more pronounced. Following a challenge with myelin debris, CE, DAGs, and triacylglycerols were upregulated in the *TREM2* KO and *PLCG2* KO cells compared with WT cells. This highlights the role of impaired TREM2 and PLCγ2 signaling in neutral lipid accumulation and, potentially, excessive LD formation. Reciprocally, it also reinforces the notion that TREM2 and PLCγ2 are involved in the same intracellular signaling cascade, and when activated, inhibit neutral lipid and potentially LD accumulation (139).

While excessive LD accumulation is associated with microglial dysfunction in several models, emerging work suggests that LD formation may serve a protective, adaptive role under specific physiological stresses. In models of demyelination, TREM2-dependent LD biogenesis supports efficient lipid storage and promotes remyelination, indicating that transient LD formation can buffer phagocytosed lipids and sustain microglial function during repair (140). TREM2 may help facilitate this balance by coordinating lipid uptake and intercellular transfer through ApoE-bound lipoproteins. Indeed, TREM2 directly differentially binds to ApoE isoforms (19, 141) and CLU, promoting lipoprotein internalization and metabolic shifts in microglia. Disruption of this axis—as seen in TREM2 loss-of-function mutations—impairs cholesterol efflux and may compromise lipid detoxification pathways that underpin horizontal lipid flux and metabolic coupling between neurons and glia (142). Moreover, this pathway is increasingly viewed as a mechanism for lipid recycling in neurodegeneration, wherein neurons offload damaged or excess lipids onto glia—particularly astrocytes—for metabolic clearance (143). Whether similar pathways exist for microglia uptake, especially in disease contexts, remains an open question but may plausibly depend on functional TREM2-ApoE interactions. One intriguing possibility is that structural or dynamical differences between TREM2 variants—particularly those involving disease-associated mutations—alter receptor conformation, ligand binding kinetics, or downstream signaling thresholds, thereby modulating microglial responses to lipid cues in a mutation- and context-specific manner. Elucidating these biophysical properties could provide a mechanistic framework for reconciling variant-specific phenotypes and identifying druggable surfaces for selective TREM2 modulation to restore microglial lipid metabolism.

### Lipoprotein lipase (LPL)

LPL has emerged as another key modifier of microglial lipid processing. Though not a classical immunoreceptor, LPL is frequently coexpressed with TREM2 and ApoE in DAMs and plays a key role in coordinating lipid uptake and immunometabolic adaptation (Fig. 3). Notably, LPL deficiency alone is sufficient to induce LD accumulation, impair lipoprotein uptake, shift substrate preference toward glycolysis, and suppress cholesterol efflux (144)—a phenotype reminiscent of TREM2 dysfunction. In addition to regulating metabolism, LPL has been shown to increase microglial phagocytosis of Aβ in rodent models of amyloidosis (27). Complementary findings indicate that upregulation of LPL during metabolic reprogramming can actively promote Aβ clearance. Reciprocally, deletion or inhibition of the glycolytic enzyme hexokinase 2 in microglia enhances lipid metabolism, increases LPL expression, and boosts ATP production, driving more efficient phagocytosis and clearance of Aβ plaques (145). Interestingly, recent studies have also shown that in response to antiamyloid immunization in AD patients, there is an upregulation of Aβ-clearing microglia, characterized by TREM2, ApoE, and LPL, supporting the notion LPL and TREM2 work synergistically in some way, and that LPL-expressing DAMs are an adaptive cell population (146).

These findings raise the possibility that variability in LPL expression, whether due to transcriptional rewiring, genotype, or environmental and pathological stressors, could modulate the impact of TREM2 loss on lipid handling and help explain the divergent outcomes observed across disease models and mutations. For instance, as summarized in a recent review, metabolic inputs such as dietary and endogenous fructose, as well as pathological insults like Aβ exposure, have been implicated in driving microglial lipid stress responses and metabolic reprogramming (22), processes associated with increased LPL expression in DAMs actively engaged in Aβ phagocytosis. Furthermore, recent transcriptome-wide association studies identified LPL expression in the hippocampus as a key determinant of cognitive function, with lower LPL levels correlating with impaired learning, memory, and synaptic signaling pathways (147). Although the cellular sources of hippocampal LPL were not defined, it is highly likely that a significant portion of the LPL signal could derive from microglia, particularly within the context of aging or neurodegenerative stress, where LPL-expressing microglial subpopulations are elevated. Together, these findings highlight LPL not only as a lipid-processing enzyme but also as a dynamic metabolic sensor that integrates environmental cues, immune activation, and lipid clearance capacity in DAMs.

Within microglia, LPL activity is dynamically regulated to integrate lipid uptake with immunometabolic adaptation. Similar to peripheral tissues, microglial LPL function is shaped by both cofactor input and inflammatory signaling. The adipokine Angiopoietin-like 4 (ANGPTL4), upregulated in aging and neurodegeneration, potently inhibits LPL activity and has been shown to drive LD accumulation in microglia by impairing TG hydrolysis and lipid clearance (148). Conversely, ApoC2 serves as an obligate cofactor for LPL activation, and the relative balance between ApoC2 and ANGPTL4—or representative peptides and fragments of these proteins, respectively (149)—may finely tune lipid uptake efficiency and LD turnover in microglia (150).

Beyond cofactor modulation, LPL activity is shaped by its anchoring to the microglial cell surface, likely via interactions with heparan sulfate proteoglycans (HSPGs), analogous to mechanisms established in peripheral tissues, though the role of such scaffolding mechanisms in microglial lipid handling remains to be elucidated. Further, while glycosylphosphatidylinositol-anchored HDL-binding protein 1 is essential for the transport, stabilization, and function of LPL at capillary surfaces in peripheral tissues and brain endothelium, it remains unclear whether analogous mechanisms govern LPL localization and activity within microglial cells. Proper folding, maturation, and stabilization of LPL are similarly critical for maintaining lipid homeostasis in microglia. Although post-translational regulation of LPL has been extensively characterized in peripheral tissues, comparable mechanisms likely operate within the CNS. Notably, the chaperone protein lipase maturation factor 1 (LMF1) is required for the post-translational maturation of LPL dimers, and mutations in LMF1 impair LPL-mediated TG metabolism, leading to severe hypertriglyceridemia (151, 152). Large-scale single-cell transcriptomic datasets, including Tabula Muris Senis (153), demonstrate detectable LMF1 expression in microglia, suggesting that LPL folding and stabilization through LMF1 may be essential for sustaining lipid uptake, clearance, and metabolic adaptation in microglial cells.

These mechanisms further intersect with ApoE biology, as ApoE-containing lipoproteins can modulate LPL activity, substrate access, and receptor interactions in an isoform-dependent manner, thereby influencing lipid processing in disease contexts. Using native human VLDL particles, Whitacre *et al.* demonstrated that ApoE content inversely correlates with LPL-mediated TG hydrolysis, with ApoE4 exerting the strongest inhibitory effect, ApoE3 having an intermediate effect, and ApoE2 showing minimal inhibition (154). Importantly, they controlled for ApoC2 levels, confirming that the native VLDL particles used in their assays contained sufficient endogenous ApoC2 to fully support LPL activation. Thus, the observed differences in lipolysis rates were not attributable to variations in classical cofactor availability but rather to differences in ApoE content. Further supporting this conclusion, exogenous addition of ApoE to ApoC2–containing VLDL particles dose-dependently suppressed LPL activity, establishing ApoE, and particularly ApoE4, as a direct and isoform-sensitive negative regulator of LPL-mediated TG hydrolysis. Their novel ex vivo plasma-like system revealed that even small differences in ApoE levels on VLDL particles significantly altered lipolysis rates, independent of TG content. Together, these findings position ApoE not only as a key determinant of immunometabolic adaptation but also as a dynamic modulator of LPL activity under physiological conditions alongside classical cofactors like ApoC2. Although ApoE-containing VLDL particles are not typically present in the brain, these findings underscore the need to determine whether ApoE4-containing CSF-Lps can inhibit LPL activity and subsequently modify microglial metabolism and function and AD risk.

Given the central role of LPL in lipid clearance and metabolic reprogramming, disruptions in LPL activity—whether through cofactor imbalance, mutations, inflammatory signaling, or ApoE isoform effects—may represent a convergence point for neurodegenerative pathologies. Recent studies have identified pathogenic LPL mutations that impair protein folding, dimerization, or secretion, such as the novel DNA-level mutations c.347G>C and c.472T>G (155) and the A98T (c.292G>A) variant (156), which cause severe hyperlipidemia and hyperlipoproteinemia, respectively, and highlight the critical importance of LPL activity for systemic lipid homeostasis. Although direct studies of the effects of LPL mutations in microglia are lacking, these findings suggest that even subtle genetic impairments in LPL function could disrupt lipid handling in the CNS and exacerbate neurodegenerative processes (157). Thus, understanding how microglial LPL activity is tuned by both intrinsic and extrinsic factors may offer new opportunities to modulate lipid handling and inflammation in neurodegenerative disease. Targeting LPL-mediated pathways represents a promising strategy to restore microglial homeostasis and mitigate AD risk and progression.

Beyond its role in hydrolyzing TG-rich lipoproteins, LPL also acts as a bridging molecule, tethering lipoproteins to heparan sulfate proteoglycans and receptors such as LDL receptor-related protein 1 (LRP1). Through these interactions, LPL enables receptor-mediated lipid internalization and coordinates metabolic reprogramming in phagocytic and DAM.

### Lipoprotein receptor-related protein 1 (LRP1)

Apart from its role in bridging-mediated uptake, LRP1 is a multifunctional scavenger and signaling receptor that plays a prominent role in microglial lipid handling, endocytosis, and inflammatory signaling. Expressed at high levels in microglia and other brain cell types, LRP1 binds diverse ligands, including lipoproteins, apolipoproteins, Aβ, and Tau, and modulates processes ranging from phagocytosis to nuclear receptor signaling. Notably, LRP1 signaling outcomes are modulated by its coreceptors; depending on the context, LRP1 interaction with partners such as TLR4 can promote proinflammatory pathways, whereas pairing with other microglial receptors (e.g., *N*-methyl-d-aspartate receptor [NMDA-R]) can drive anti-inflammatory signaling.

Several recent studies have revealed direct roles for LRP1 in modulating microglial lipid metabolism and inflammatory signaling cascades relevant to AD. For instance, exposure to extracellular tau increased LRP1 expression in microglia and elicited a proinflammatory response that was independent of NMDA-R signaling (158). While the authors proposed that TLR4 may act in concert with LRP1 in this process, further studies are needed to clarify the precise receptor mechanisms involved. Chen *et al.* (159) further demonstrated that physical exercise enhances LRP1 expression in microglia and peripheral immune cells in the 5xFAD AD mouse model, suggesting that exercise may modulate microglial immune function—and potentially lipid metabolism—through *LRP1* upregulation. Although this study did not directly examine lipid processing, the link between exercise and microglial lipid metabolism is supported by recent work showing that high-intensity interval training alters LD number and size in hippocampal microglia, indicating that exercise can directly influence microglial lipid handling in vivo (13). Together, these findings suggest that exercise-induced *LRP1* upregulation may contribute to broader microglial lipid metabolic remodeling in disease states.

Additional evidence for lipid-linked LRP1 function comes from studies implicating LRP1 in NF-κB–regulated inflammatory signaling. In BV-2 microglial cells, the bioactive plant compound Andrographolide (Andro) attenuated Aβ-induced inflammation by activating the lipid-sensing nuclear receptor peroxisome proliferator-activated receptor γ through an LRP1-mediated pathway, ultimately suppressing NF-κB signaling (160). Similarly, treatment with the metabolite ginsenoside compound K reduced Aβ42-induced inflammation in BV-2 microglia by upregulating LRP1, which in turn suppressed NF-κB signaling and inflammatory cytokine production (161). These findings link LRP1 to innate immune regulation and microglial responses to metabolically active compounds. A complementary mechanism involves a synthetic peptide derived from the cellular prion protein, which has been shown to suppress LPS-induced inflammation in BV-2 microglia through LRP1- and NMDA-R-dependent mechanisms (162). This anti-inflammatory signaling occurs within lipid rafts—cholesterol-rich membrane microdomains—where cellular prion protein laterally associates with the LRP1/NMDA-R complex (163), facilitating LRP1-mediated signaling and further supporting its role in lipid-linked immune modulation.

Finally, LRP1 also plays a direct mechanistic role in microglial clearance of lipid-rich cargo. Zhai *et al.* (164) demonstrated that repetitive trans-spinal magnetic stimulation promoted microglial clearance of myelin debris after spinal cord injury via an LRP1-dependent pathway. Inhibition of LRP1 with receptor-associated protein abrogated these effects, establishing LRP1 as a functional mediator of lipid-rich debris clearance in microglia. Further supporting the role of LRP1 in lipid-linked signaling, activating LRP1 using the ApoE-mimetic peptide COG1410 suppressed neuroinflammation and oxidative stress via inhibition of the thioredoxin-interacting protein and NLR family pyrin domain containing 3 signaling pathway and promoted anti-inflammatory microglial polarization, in a cerebral ischemia/reperfusion model (165). This pathway integrates lipid receptor signaling (through LRP1) with oxidative stress responses (through thioredoxin-interacting protein), which are known to impair lipid processing in microglia and promote LD accumulation and dysfunctional clearance (13). As such, this study underscores the role of LRP1 in linking lipid sensing to oxidative stress-driven shifts in microglial phenotype. Collectively, these studies support a multifaceted role for LRP1 in microglia: as a lipid receptor mediating uptake of lipoproteins and myelin debris, a modulator of nuclear receptor and inflammatory signaling, and a key integrator of metabolic cues with microglial functional states.

### Low-density lipoprotein receptor (LDLR)

Beyond LRP1, the LDL receptor (LDLR) also contributes to microglial lipid metabolism and immune regulation. LDLR binds ApoE-containing lipoproteins and other lipid-rich ligands and mediates their uptake, playing a central role in cholesterol trafficking and clearance within the brain. Because lipidation of ApoE is required to expose its LDLR-binding domain, recent studies have focused on lipidated forms of ApoE to accurately model receptor interactions, highlighting striking isoform-dependent differences in LDLR-lipidated ApoE interactions. For example, using surface plasmon resonance and homogeneous time-resolved fluorescence assays, Guo *et al.* demonstrated that lipidated ApoE2 exhibits dramatically impaired binding to LDLR compared with ApoE3 and ApoE4 (166). Isoform-dependent differences in LDLR-mediated uptake were further confirmed in microglial cultures, where heparin treatment—known to block ApoE interactions with HSPGs—attenuated lipidated ApoE3 and ApoE4 uptake but was much less effective for lipidated ApoE2, consistent with its reduced LDLR binding affinity. In addition, genetic deletion of *LDLR* markedly reduced lipidated ApoE3 and ApoE4 internalization, and coincubation with the soluble extracellular domain of LDLR suppressed uptake of these isoforms. Of note, while early ApoE internalization appeared to involve multiple receptors, they further found that LDLR became the dominant pathway for ApoE uptake over longer exposure periods.

Importantly, reduced LDLR binding by lipidated ApoE2 was not deleterious; rather, it appeared protective against lipid-associated pathology. Guo *et al.* demonstrated that LDLR-mediated uptake of lipidated ApoE3 and ApoE4 delivered substantial amounts of CEs into microglia, whereas lipidated ApoE2 resulted in markedly lower CE accumulation, consistent with its impaired LDLR binding and internalization. These lipid challenges provoked transcriptional changes in microglia, including upregulation of cholesterol efflux genes and increased secretion of inflammatory cytokines (especially for lipidated ApoE4). These data underscore the role of LDLR as a conduit for isoform-specific lipid uptake in microglia, linking ApoE-lipid complexes to inflammation and lipid stress. Moreover, despite the similar LDLR binding affinities of lipidated ApoE3 and ApoE4, lipidomic analyses revealed that endogenous ApoE4 particles were enriched in CEs containing ARA (CE(20:4)), a highly peroxidizable species that promotes lipid aggregation and oxidative damage. These findings suggest that the pathogenic outcomes of ApoE-driven lipid uptake depend not only on receptor binding affinity but also critically on the lipid cargo composition. Supporting this, replacing CE(20:4) with a less peroxidation-prone oleic acid-containing CE(18:1) reduced oxidative markers such as lipofuscin and prevented aggregation in lysosomes, confirming that the lipid cargo’s susceptibility to peroxidation crucially determines its pathogenicity.

Further linking lipid composition to neurodegenerative stress, Guo *et al.* showed that exposure to tau fibrils exacerbated lipofuscin accumulation in iNeurons (a human iPSC-derived neuron), an effect that was fully rescued by the ApoE3-Christchurch variant, which carries a mutation (R136S) that diminishes receptor binding—much like ApoE2, thereby mimicking its protective effects. These findings suggest that excessive LDLR-mediated uptake of peroxidizable lipids by microglia may sensitize neurons to tau-induced oxidative stress. Furthermore, while ApoE2 particles were more prone to aggregation in vitro than ApoE3 or ApoE4, their poor binding to microglial LDLR may protect neurons by limiting receptor-mediated lipid uptake and subsequent peroxidative stress. Notably, these aggregation assays were performed using artificially lipidated ApoE particles containing equivalent CE(20:4) content across isoforms, indicating that the greater aggregation propensity of ApoE2 at neutral pH arises from intrinsic structural properties rather than lipid cargo differences. These results identify LDLR as a critical mediator of isoform-specific microglial lipid uptake and oxidative damage pathways in the brain and suggest that attenuating LDLR interactions with peroxidizable ApoE-lipid complexes may represent a therapeutic strategy to reduce lipid-mediated toxicity in AD.

Beyond the mechanistic evidence from Guo *et al.*, multiple in vivo studies reinforce the importance of LDLR in microglial lipid metabolism and neuroinflammatory regulation. In a tauopathy model, Shi *et al.* (167) showed that LDLR overexpression reduced brain ApoE levels, preserved myelin integrity, and attenuated microglial activation, ultimately limiting tau pathology. These findings support a protective role for LDLR in maintaining lipid homeostasis and modulating microglial reactivity in vivo and suggest that boosting LDLR may help mitigate neuroinflammatory damage by promoting clearance of excess ApoE and lipid cargo. Complementing this functional evidence, transcriptomic analyses identified LDLR among lipid metabolism genes enriched in microglial caveolae—specialized plasma membrane domains associated with reactive oxygen species production—highlighting a potential structural link between LDLR localization and inflammatory signaling pathways relevant to AD (168).

Most recently, Kaye *et al.* (169) demonstrated that *LDLR*-KO microglia, especially under a high-fat diet to induce hypercholesterolemia, accumulate excess cholesterol and exhibit a compensatory shift in lipid metabolism: genes for cholesterol efflux are upregulated, whereas those for cholesterol synthesis are downregulated. This suggests microglia lacking LDLR attempt to expel excess cholesterol and curb its production, indicating that LDLR normally helps prevent lipid overload in microglia by mediating uptake and clearance of ApoE-lipoprotein complexes. LDLR-deficient microglia under a high-fat diet also developed a proinflammatory lipidomic signature, with elevated levels of bioactive lipids in their membranes that induce inflammation and mitochondrial dysfunction. Notably, principal component analysis of the microglial lipidome revealed a clear separation by LDLR genotype and diet, underscoring that loss of LDLR profoundly alters microglial lipid composition. In the context of AD, these changes had functional consequences: *LDLR*-KO microglia displayed impaired clustering around Aβ plaques, reduced expression of DAM markers, and contributed to less compact plaque structures, particularly under hypercholesterolemic conditions. Mechanistically, this may result from failed clearance of ApoE-Aβ complexes—LDLR-deficient mice showed elevated brain ApoE levels, especially around plaques and under hyperlipidemic conditions, potentially crowding out or competing with Aβ for LDLR-mediated clearance pathways.

Together, these studies paint a coherent picture in which LDLR is a double-edged sword: while it is essential for maintaining microglial lipid homeostasis and facilitating clearance of ApoE-Aβ complexes, excessive or dysregulated LDLR activity can drive lipid overload and downstream dysfunction. In particular, sustained LDLR-mediated uptake of ApoE-lipid particles—especially those enriched in peroxidizable species or containing ApoE4—can overwhelm the endolysosomal system, leading to cholesterol accumulation, lysosomal stress, and oxidative byproducts such as lipofuscin. These effects may propagate neurodegenerative changes both within microglia and in neighboring neurons. Thus, while baseline LDLR function is protective, as demonstrated by reduced brain ApoE levels and preserved myelin integrity under LDLR overexpression, attenuating LDLR-ApoE interactions in hyperlipidemic- or ApoE4-rich contexts may help limit lipid-driven toxicity. Collectively, the evidence supports a model in which both deficient and excessive LDLR activity can disrupt microglial function and lipid metabolism, with outcomes shaped by lipid composition, diet, ApoE isoform, and disease context, including AD.

In summary, recent studies have extensively investigated microglial lipid and lipoprotein receptors, focusing on their impact on lipid processing and downstream effector functions. These investigations aim to determine whether activating or inhibiting these receptors can enhance microglial functions, such as increased phagocytosis and reduced LD accumulation, to mitigate AD risk. While several insights, particularly from rodent models of AD, have been promising, the findings remain inconsistent and context dependent. This inconsistency may stem from the complex and underappreciated interactions between these receptors (Fig. 3). Therefore, further studies are needed to elucidate the interactions between microglial lipoprotein receptors and their ligands. Given the challenges of empirically defining complex receptor interactions, initial studies using high-powered molecular simulations could help define specific interactions, which can then be studied more comprehensively through biophysical and biochemical analyses. A significant roadblock to this approach is the lack of lipidated apolipoprotein structures, which are essential for simulating interactions and narrowing down areas for further study, highlighting the need for progress in this area. Despite these challenges, targeting microglial lipoprotein receptors remains a promising strategy for improving microglial functions and reducing AD risk.

## Summary, Future Directions, and Conclusions

Our understanding of AD and its complex neuropathogenesis is continually evolving, with recent advancements highlighting the critical role of brain and microglia-specific lipid metabolism. Overall, recent studies have highlighted the association between increased brain TGs and cholesterol, yet reduced FAs, with AD onset and progression (Table 1). Moreover, recent studies have shown that associations with specific apolipoproteins may determine whether alterations in lipid and cholesterol abundance are also associated with disease prevention or protection (Table 1). It is also likely that elevated TG and cholesterol levels in AD are due to alterations in cell composition and phenotype, particularly in microglia.

**Table 1:** Specific functions of lipid, lipoprotein, and apolipoprotein species on microglia and their potential as targets for neurodegenerative disease

Lipid and lipoprotein species	Function	Disease model/clinical interventions
PLs	• Integral to cell membrane structure • Enable signaling transduction and neuronal-glial communication • PS acts as an “eat-me” signal for microglia (35) • PLs are generally depleted in the AD brain, but C18:0 and DHA-containing PS may be increased	• *LPCAT3* depletion in murine microglia (App^NL-GF^) promotes microglial phagocytosis, facilitates de novo lipogenesis, and protects against oxidative damage (45) • Deleting microglial Mertk prevents PS sensing and inhibitory synapse elimination by microglia (170) • PL supplementation (e.g., PC-DHA) may improve outcomes in rodent models of AD (43)

Saturated FAs (SFAs)	• Components of PLs, sphingolipids, and TGs • Saturated LCFAs are associated with an increased risk of progressing from MCI to AD (171) • SFAs are relatively enriched in microglia (172) • SFAs may polarize microglia toward a more inflammatory state, which may impact neuronal function (173)	• High intake of SFAs may increase AD risk (174) • Strategies that reduce dietary SFAs may improve microglial function and AD outcomes

MUFAs	• Components of PLs, sphingolipids, and TGs • MUFAs may attenuate microglial inflammation (175, 176) in vitro	• Stearoyl-CoA desaturase (SCD) inhibitor prevented MUFA production, restored hippocampal function, and dampened microglial activation in vivo (177)

PUFAs	• Component of PLs, sphingolipids, and TGs • LC-PUFAs are precursors to inflammatory modulators made by microglia • Supplementation with DHA in vitro attenuates inflammation, increases microglial PS, and reduces LD size (44)	• PUFA supplementation (EPA, C20:5, and DHA) may reduce neuroinflammation, Aβ accumulation, and cognitive decline (178) • PUFA supplementation and FA conversion (via FAT-1) may reduce microglia number and improve neurodegeneration (179) • FASN inhibitor (CMS121) increased PUFAs and reduced memory decline in an AD mouse model (APPswe/PS1ΔE9) (180)

Cholesterol	• Integral to myelin and membrane integrity • Provides trophic support to glial cells • Microglia accumulate CE in LDs. CE is associated with impaired phagocytosis (170) • LPS and ApoE4 increase *CH25H* and CH25 (61, 62)	• Genetic depletion *CH25H* in a rodent model reduced phosphorylated tau (56). • Cholesterol-lowering agents (e.g., statins) may not only lower amyloid-β levels but also impair the BBB permeability (181)

Sphingolipids	• Sphingolipid are involved in lipid signaling and signal transduction in microglia • Cer is increased in aging and AD • Cer accumulation leads to microglial activation (75) • Cer (C24:1) is synthesized by microglia and is enriched in microglia-derived EVs from AD brains (76) • S1P regulates neuron-microglia communication	• Sphingosine-1-phosphate receptors 1 and 2 (S1PR1, S1PR2) inhibitor (fingolimod) partially resolved inflammation in microglia (69) • The S1PR1 antagonist, ponesimod, prevents Aβ-induced activation of microglia (82)

TGs	• Serve as energy substrates for energy production during high metabolic demand and stress • Microglia accumulate TGs in AD models (6, 14)	• Acyl-CoA synthetase long-chain family member 1 (ASCL1) inhibitor (Triacin C) prevents LD accumulation in ApoE4 microglia • Inhibition of DGAT2 prevents LD formation, enhances microglial phagocytosis, and reduces amyloid-β load (71)

ApoE	• Mediates lipid and cholesterol transport between neurons and glial cells • Regulates LD biogenesis and composition • Increased expression in DAMs • Exposure to ApoE4 leads to LD formation and immunometabolic polarization (105, 106)	• Adeno-associated virus-based gene therapy to express the ApoE2 allele in the brains of homozygous ApoE4 individuals might slow disease progression [LX1001] • Anti-ApoE4 antibodies may reduce amyloid-β plaque load and modify glial responses (182)

ApoA1	• Liver derived • Promotes cholesterol efflux in macrophages • May improve microglial phagocytosis (113)	• ApoA1 mimetic peptide (5A) promotes cholesterol efflux, improved remyelination, and increased uptake of myelin-derived lipids (112)

SPP1	• Involved in the activation of microglia • Increased expression in DAMs • A microglia-derived extrinsic signal to mediate crosstalk between glial cells and neurons	• Anti-SPP1 antibody inhibited proinflammatory microglia responses and reduced amyloid-β plaque pathology in an AD mouse model (5xFAD) (183)

To better address the multifaceted nature of AD, future research must overcome several key challenges. First, there is a dire need for technological advancements that enable precise characterization of brain lipidation status and CSF-Lp composition. Collection procedures that allow for studies in different biofluids, such as brain interstitial fluid, would enable us to differentiate between various lipoproteins, trace their origins, and elucidate their specific actions within the CNS. Understanding these intricate dynamics is crucial for accurately defining their contributions to AD pathology. A perhaps loftier goal would be leveraging our understanding of brain lipoprotein subspecies to bolster specific populations that target dysregulated cells and areas of the brain to restore functionality and improve AD outcomes.

An emerging strategy to improve AD neuropathogenesis has been targeting microglial TG synthesis to deplete LD accumulation and to restore cellular functionality, such as phagocytosis of amyloid and cellular debris. While the findings from these studies are promising, a more comprehensive understanding of microglial lipid and lipoprotein processing in various disease states and contexts is necessary to carefully consider inhibiting LD accumulation. In addition, there are several other aspects of microglial lipid metabolism that provide potential targets to improve AD pathology. Namely, reducing microglial CE accumulation to improve and restore phagocytic capacity and preventing Cer synthesis to reduce the biosynthesis of EVs that would otherwise propagate the spread of Aβ and tau (Table 1).

Given the complexity of lipid interactions and their systemic effects, computational approaches, such as molecular simulations, offer a promising avenue for overcoming these challenges. These simulations can streamline research by modeling lipid-protein interactions and predicting how changes in lipid composition affect microglial function and AD progression. Employing such methodologies could also accelerate the design of novel pharmacological interventions tailored to effectively modulate lipid metabolism.

However, even as we strive for advanced solutions, practical strategies such as dietary and lifestyle modifications should not be overlooked. Reducing circulating lipids through these means may provide a valuable and accessible approach to alleviating some of the burden of AD. Additionally, specific interventions targeting the accumulation of long-chain Cers and TGs, as well as utilizing DHA for its signaling properties, present focused strategies that warrant further exploration and development (Table 1). We can also not rule out the contribution from other metabolic pathways (e.g., glycolysis) to lipid synthesis, such as de novo lipogenesis. Given the exciting data linking therapeutics that modify systemic glucose homeostasis, insulin secretion, and body weight (e.g., glucagon-like peptide-1 agonists) to reduce AD onset, it will be important to determine how these factors modify microglial lipid accumulation and function.

In conclusion, a comprehensive strategy integrating technological innovation, molecular simulations, and practical lifestyle interventions holds promise for significantly advancing our ability to modulate lipid metabolism in AD. By targeting the underlying lipid dysregulation, we can potentially improve microglial function and alter disease outcomes, offering hope for treatments that broadly impact individuals with or at risk of AD in the future.

## Conflict of interest

The authors declare that they have no conflicts of interest with the contents of this article.
